# Unequal Recovery? Federal Resource Distribution after a Midwest Flood Disaster

**DOI:** 10.3390/ijerph13050507

**Published:** 2016-05-17

**Authors:** Cristina E. Muñoz, Eric Tate

**Affiliations:** Department of Geographical & Sustainability Sciences, University of Iowa, Iowa City, IA 52242, USA; eric-tate@uiowa.edu

**Keywords:** property acquisition, buyouts, environmental justice, social vulnerability

## Abstract

Following severe flooding in 2008, three Iowa communities acquired over 1000 damaged properties to support disaster recovery and mitigation. This research applies a distributive justice framework to analyze the distribution of disaster recovery funds for property acquisition. Two research questions drive the analysis: (1) how does recovery vary by acquisition funding source; and (2) what is the relationship between recovery and vulnerable populations? Through spatial econometric modeling, relative recovery is compared between two federal programs that funded the acquisitions, and across socially vulnerable populations. The results indicate both distributive and temporal inequalities in the allocation of federal recovery funds. In particular, Latino and elderly populations were associated with lower recovery rates. Recommendations for future research in flood recovery and acquisitions are provided.

## 1. Introduction

In June 2008, the Midwestern U.S. received over 12 inches of rainfall as several successive storm systems impacted the region [1]. The heavy sustained rainfall fell on previously saturated land and increased the magnitude of the flood [2]. Eighty-five out of ninety-nine counties in Iowa were declared a Federal Disaster Area, making it the largest flood event since the Flood of 1993. Following the 2008 flood, the Eastern Iowa cities of Cedar Rapids, Palo, and Iowa City were among several that used federal disaster funds to acquire nearly 1500 flood-affected properties.

Federally-funded property acquisition has played an increasing role in the mitigation of, and recovery from U.S. flood disasters [3,4]. As part of the acquisition process, homeowners voluntarily sell their flood-damaged properties at pre-flood market values. Federal programs for property acquisition include the Hazard Mitigation Grant Program (HMGP), the Community Development Block Grant (CDBG), and the Flood Mitigation Assistance Program. From 1998 to 2003 alone, completed HMGP acquisitions totaled to 10,246 properties [5]. Future flood losses are avoided when acquired land is permanently repurposed to open-public green space [6]. Doing so can also enhance critical ecosystems and provide cultural services through recreational space [7]. Also, buyout programs provide an opportunity for individuals to recover by offering pre-flood market values for their flooded homes.

There are also limitations to voluntary buyouts. Previous research has found that lack of trust between program managers and low-income and elderly communities led to difficulties implementing the voluntary aspect of property acquisition [6,8]. Meanwhile, low-income homeowners may not be adequately compensated [8]. As property acquisition becomes an increasingly important tool for recovery and mitigation, it is important to better understand its relationship with disaster recovery.

This research investigates recovery facilitated by federal funds for property acquisition, from the perspective of social equity. Inequities in resource distribution or social opportunities can produce recovery outcomes that disproportionally favor well-off groups over those in unfavorable and vulnerable conditions [9,10,11,12]. We adopt a justice framework to evaluate the degree to which equitable recovery was achieved. Two research questions drive the analysis:

(1) how does recovery vary by acquisition funding source; and
(2) what is the relationship between recovery and vulnerable populations?

The remainder of this paper proceeds as follows. Section 2 reviews current understanding of flood recovery and equity, and Section 3 discusses federal programs that fund property acquisitions. Section 4, Section 5 and Section 6 describe the methods, results, and discussion of the key findings. Section 7 concludes with future research needs.

## 2. Justice and Flood Recovery

Before the 1990s, disaster studies primarily conceptualized recovery as a predictable sequential process composed of multiple stages that eventually led back to “normalcy” [13]. However, bringing communities back to their pre-disaster stage could recreate the vulnerable conditions that led to the disaster [14,15], and exacerbate marginalization and inequalities during the recovery process [10,13,16]. Recent conceptualizations of recovery acknowledge that individuals experience recovery at different rates and bear different outcomes. Thus, recovery can be defined as “the differential process of restoring, rebuilding, and reshaping the physical, social, economic, and natural environment” [17] (p. 237). The differential aspect refers to unequal social and economic access to resources that influences recovery outcomes. From this perspective, the distribution of property acquisition resources may be a differential process. In particular, the allocation of funds includes procedural and distributive justice dimensions.

Procedural justice concerns inequities in the meaningful involvement of socially vulnerable populations. The distribution of federal funds is often a political process [11,18], which potentially favors those already in power both at the state and community level [19,20]. Federal program objectives, method of decision making, community engagement, and eligibility prerequisites all have a bearing on how communities will recover [21]. Bureaucratic obstacles and language barriers often prevent recent migrants from securing disaster funds during recovery [22,23]. Without embedding procedural justice in the decision-making process for post-disaster resource allocation, vulnerability reduction and recovery outcomes may be inequitable [9].

Equitable distribution of resources is another an important characteristic of successful recovery [24]. Inequitable distribution during flood recovery has been found to impact the most socially vulnerable, including minorities, female-headed households, low-income households, and the elderly [25]. Race and income could also influence a person’s ability to access recovery assistance [26,27]. Specifically, African-Americans and Latinos have incurred greater damage, had longer periods of temporary housing, and have been less likely to secure adequate resources from flood insurance and the federal government during recovery [28,29]. In Hurricane Katrina, waivers in recovery funding provisions were particularly unfavorable to socially vulnerable populations [30]. The focus on asset protection in flood recovery programs impairs recovery for vulnerable populations such as renters and low-income families. Furthermore, research investigating fairness in flood risk management in Europe concluded that current priority given to cost-benefit analysis in decision making for flood protection “fall short of being fair from either a vulnerability or equality perspective” [31] (p. 374). Equitable distribution of recovery funds requires further investigation [32,33].

## 3. Case Study

### 3.1. Federal Programs for Property Acquisition

The two leading U.S. federal sources that fund buyouts are the HMGP and the CDBG programs. The U.S. Federal Emergency Management Agency (FEMA) administers HMGP. FEMA has had voluntary buyout authority since the 1980s, but legislative amendments in 1993 led to large-scale opportunities for property acquisition [34]. The primary goals of HMGP are to reduce future hazard risk to people and property, and to implement long-term hazard mitigation measures in the recovery process [35]. To be eligible for HMGP buyout funds, properties must either be located in the Special Flood Hazard Area (e.g., 100-year flood zone) or be cost-effective based on a benefit-cost analysis [36]. Properties acquired with HMGP funds are deed restricted against structural improvements, and thus are limited to future use as open space, recreation, or natural floodplains [34].

CDBG is administered by the U.S. Department of Housing and Urban Development, which primarily functions to address urban poverty and redevelopment. CDBG has three national objectives: (1) benefit low-to-moderate income persons; (2) aid in the elimination of urban blight; and (3) meet urgent development needs that threaten community welfare. Due to the third objective, CDBG has provided approximately $40 billion in disaster relief funds to assist with recovery efforts since 1993 [37]. CDBG disaster relief goals are to assist in community development and social aspects of recovery rather than mitigation [38]. Outside of the Special Flood Hazard Area, CDBG property acquisition is not deed restricted and allows structural improvements. CDBG is therefore an alternative to HMGP when construction of floodwalls or housing is desired on the acquired land.

Both HMGP and CDBG acquisitions must satisfy duplication of benefits provisions, which become salient when assistance from more than one source is used for the same purpose or activity [39]. Consider the example of a $60,000 home damaged by flooding, where the homeowner receives a $20,000 loan from Small Business Administration (SBA) to make repairs. If the owner later decides to participate in a property acquisition program, the $20,000 in improvements to the flooded home would not contribute to the assessed value when determining the acquisition cost. Any disaster relief or recovery project must be evaluated for duplication of benefits and other environmental regulations before federal funds can be distributed to individuals [40].

### 3.2. Study Area

Three Eastern Iowa cities compromise our study area; Cedar Rapids, Palo, and Iowa City. These cities vary in size and in population demographics. Table 1 outlines some characteristics of the cities [41].

Collectively, the cities acquired 1461 residential properties after the 2008 floods. Table 2 displays descriptive information for the acquisition of single-dwelling residential properties. The table includes the total number of acquisitions, pre-flood appraisal multiplier set by each city, and total cost of residential acquisitions. The appraisal multiplier is determined by the assessor office of each city and approved by the city’s mayor.

In Cedar Rapids, floodwaters affected more than 14% (10 square miles) of the city, and damaged both residential and commercial areas [42]. Residential acquisitions totaled to $58.1 million, which include 1183 properties purchased at the 107% of the pre-flood value [43]. The acquisitions were grouped and prioritized into three areas defined by the city; the “Greenway”, the “Construction”, and the “Neighborhood Revitalization” areas [44]. The Greenway Area is for the development of an urban park located within the 100-year flood zone, and contains 120 HMGP and 104 CDBG acquired residential properties. The adjacent Construction Area contains 350 residential properties acquired through CDBG, and is targeted for the development of floodwalls and levees. The Neighborhood Revitalization Area contains 609 residential CDBG acquisitions, and is intended for affordable housing and economic development outside of the 100-year flood zone [44].

In Palo, all but 13 of the 423 homes sustained moderate to significant damage [45]. Through a six-month public participation planning process, Palo defined recovery goals centered around rebuilding and expanding both the commercial and residential districts [46]. Property acquisition played a small role in city recovery. Only a dozen homes were purchased with HMGP funds, assessed at 110% of the pre-flood value [47]. To redevelop the business district, over $12.1 million in SBA loans were distributed for home and business repair. Palo also used other CDBG programs to fund recovery activities unrelated to buyouts.

In Iowa City, the main recovery goal was to mitigate future floods through property acquisition [48]. The city acquired 93 residential homes in the Parkview Terrace neighborhood, purchased at 112% of the pre-flood home value. Including relocation expenses, the acquisition project was estimated to cost $22 million [49]. Iowa City purchased 46 properties through HMGP in the 100-year flood zone to achieve its non-structural mitigation objective. The remaining acquisitions were purchased with CDBG funds, and used to meet both structural and non-structural mitigation needs.

## 4. Methods

### 4.1. Input Data

Table 3 summarizes the geospatial data collected for the analysis. Flood depth grids for the 2008 peak stage were obtained from the Iowa Flood Center. Tax assessor data for the three cities include property class (e.g., commercial, residential), occupancy (e.g., single dwelling), and 2008 pre-flood assessed property values. Demographic information was acquired from the 2000 census at the block-group level. The acquisition data was collected from FEMA and the Iowa Economic Development Authority (IEDA).

The acquisition programs were still in progress at the time of our analysis. As a result, 978 of the 1183 Cedar Rapids acquisitions are reflected in our data. All 12 Palo acquisitions and 83 out of 93 Iowa City acquisitions are captured in the data, which yield a total sample size of 1073 acquisitions. We geocoded the acquired properties to the parcel centroid and joined them with tax assessed property values. The data include acquisition of both residential and commercial buildings, but only residential buildings were analyzed to facilitate comparison across all three cities.

**Figure 1:**
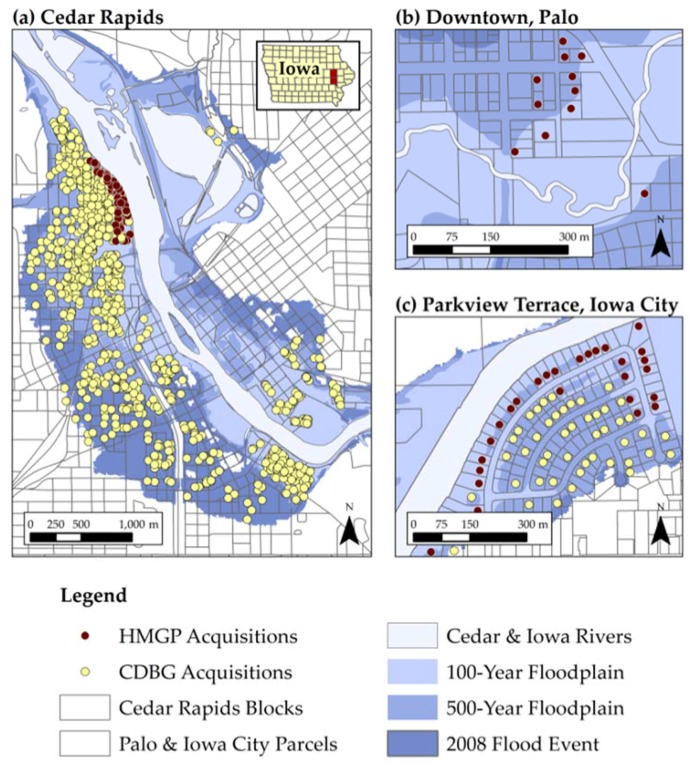
Spatial distribution of residential property acquisitions by city: (**a**) Cedar Rapids; (**b**) Downtown, Palo; (**c**) Parkview Terrace, Iowa City.

Figure 1, shows the spatial distribution of residential property acquisitions. Cedar Rapids (Figure 1a) had the largest number of acquisitions, most of which are located outside of the 100-year floodplain and purchased with CDBG funds. Within the 100-year floodplain, there are both CDBG and HMGP funded acquisitions. Palo acquisitions (Figure 1b) were funded by HMGP and were located within the 500-year floodplain. Iowa City acquisitions (Figure 1c) had residential buyouts in the Parkview Terrace neighborhood. The buyouts located within the 100-year floodplain are funded by HMGP, while CDBG funded most of the acquisitions outside the 100-year floodplain.

### 4.2. Measuring Recovery

To measure equity in the distribution of property acquisition funds, we applied a relative recovery ratio (RRR), computed as the total dollar amount received divided by the property’s 2008 pre-flooded assessed value and appraisal multiplier (Equation (1)).

(1) $$\text{RRR}=\frac{\text{AF}}{\text{AV}\times \text{AM}}$$

where acquisition funds (AF) is the total amount of dollars received for property acquisition; assessed value (AV) is the 2008 pre-flooded assessed value of the property and land combined; and appraisal multiplier (AM) represents the appraisal multiplier set by each city.

The relative measure was constructed to control for variations in home value, which is influenced by factors including the age of the structure and neighborhood amenities. We used the assessed value as a proxy for appraisal value, as have other studies [50]. The appraisal multiplier varies by city and sets the cost of each acquisition to 100% or higher of the total value of the house (Table 2). The relative recovery ratio yields a value of 1.0 when the federal funds received equal the total cost (assessed value times appraisal multiplier) of property acquisition. An RRR of less than 1.0 indicates households that received a lower amount of acquisition funds compared to the assessed home value, and *vice versa* for RRR values greater than 1.0. The measure could be greater or smaller than 1.0 due to re-appraisals and duplication of benefits among other probable causes.

The RRR metric can be used to evaluate two perspectives on distributive justice: the libertarian and the Rawls difference principles. The libertarian principle calls for the allocation of material goods to all members of society on the basis of labor and free market [51]. Under this principle, federal funds would be allocated based solely on the fair-market value of the property without regards to socioeconomic needs, and the RRR would be 1.0 for each acquisition. By contrast, the Rawls difference principle permits divergence from equal resource distribution if the inequities make the least advantaged materially better off [52]. Under this principle, acquisition monies would be preferentially allocated to low-income and socially vulnerable populations to alleviate underlying social inequalities [53,54,55]. Based on Rawlsian logic, the RRR would be expected to have values greater than 1.0 for vulnerable populations, which would represent compensation beyond the financial loss of the home.

Given the different foci of HMGP (mitigation) and CDBG (urban redevelopment), resulting differences in programmatic and procedural requirements could lead to differences in recovery rates. This leads to our first research question, how does recovery vary by federal program. We used the nonparametric Mann-Whitney *U* test for independent samples to evaluate differences in the RRR by funding program.

### 4.3. Recovery and Social Vulnerability

The second research question explores the relationship between recovery and vulnerable populations. We employed ordinary least squares (OLS) regression to test the relationship among the average RRR, social vulnerability, and flood exposure characteristics. Before applying the regression model, the individual relative recovery ratios were aggregated to the block group level by taking the average RRR value within each block group. As a result, the 1073 individual properties were aggregated to 25 block groups, representing block groups in the study area that contained at least three acquired properties.

The OLS model was tested for violations of independence, including spatial autocorrelation. Spatial autocorrelation is the coincidence of value similarity with locational similarity. If the Local Moran’s I test results were significant, we explicitly incorporated spatial autocorrelation in the model construction to reduce spatial effects on the coefficients. We employed the lag and the error autoregressive specifications [56]. The lag model incorporates spatial autocorrelation as a coefficient modifying the relationship between the dependent and independent variable, while the error model deems the autocorrelation to be in the error term.

The Lagrange multiplier statistic and the adjusted Akaike’s information criterion (AIC_c_) were evaluated to determine which econometric model best improves model fit. A decrease of AIC by three indicates a significant improvement of the model performance [57]. For both the Moran’s I and the spatial econometric modeling, the spatial weights matrix was derived using the inverse Euclidean distance spatial relationship, with row standardization and a distance band at 12 kilometers. This distance band was used to prevent acquisitions from one city being considered neighbors of acquisitions in another.

The OLS model includes demographic variables. In general, low-lying flood-prone land tends to be occupied by low-income households, racial and ethnic minorities, renters, and the elderly [9,25,58]. These populations are typically among the most vulnerable to disaster inequalities [28,29,59], and were used as the basis for the selection of independent variables in the regression model. Demographic data were obtained from the 2000 U.S. Census at the block group level, which is the lowest resolution for which both demographic and economic data are available. The American Community Survey from 2005 to 2009 was not used because their estimates are less statistically reliable at the block group scale due to smaller sample sizes compared to the decennial census [60]. 

The average flood depth for each census block group was also included in the OLS model. All three cities have substantially different median property values (see Table 1). We control for these differences in the OLS model using a dummy variable to distinguish each city. Additionally, we differentiate block groups by those that only contained HMGP or CDBG acquisitions, and those that contained both within the census boundaries. Table 4 provides the means and standard deviations (SD) of the variables considered for the OLS and econometric models.

## 5. Results

### 5.1. The Relative Recovery Ratio (RRR)

Table 5 includes the descriptive statistics for the relative recovery ratio (Equation (1)). The range of the RRR has a minimum of 0.09 and a maximum of 2.02. In other words, at least one homeowner received 9% of the property value in federal dollars, while another homeowner received more than twice the federal dollars than the total value of their home (202%). Table 5 excludes outliers based on the labeling rule for outliers [61,62]. The results of the labeling rule estimated an upper bound of RRR 2.2, so five acquisitions with RRR values ranging from 2.4 to 11.0 were excluded from the analysis. The remaining properties, with RRR between 0.09 and 2.02, have dollar amounts ranging from $3,000 to $970,000.

**Figure 2:**
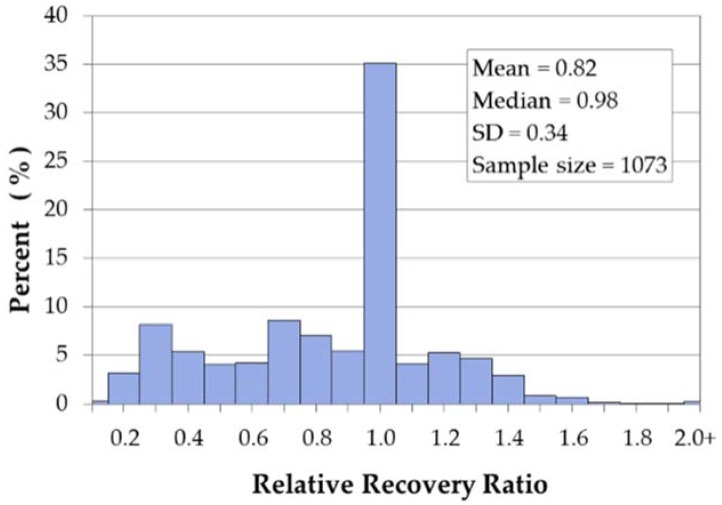
Histogram for RRR values.

Figure 2 is a histogram of the RRR values. Approximately 35% of the values fell between 0.95 and 1.05, representing homeowners that received funding amounts similar to the pre-flood value of their homes. This grouping represents the largest proportion of all acquisitions. However, the RRR metric varies from 0.09 to 2.02, indicating that some property owners received lower or much higher proportions of federal dollars compared to the assessed values of their homes. About 10% of the RRR values are 1.3 or higher and another 10% of the RRR values are 0.3 or lower. These represent extremes in the recovery spectrum. Although a few recovered more money from federal funds than their financial loss, others recovered less than 3% of their loss.

**Figure 3:**
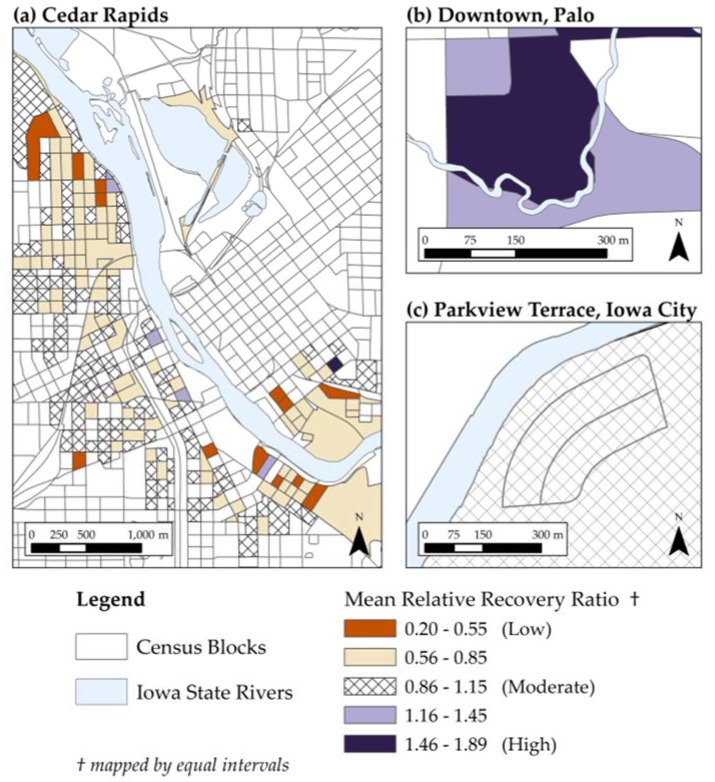
Spatial distribution of RRR values by city: (**a**) Cedar Rapids; (**b**) Downtown, Palo; (**c**) Parkview Terrance, Iowa City.

The spatial distribution of the RRR values is displayed in Figure 3. The RRR values are aggregated to the census block level and mapped using equal intervals to highlight areas of low and high relative recovery. No statistical analysis was conducted at the census block level. Blocks with mean RRR between 0.86 and 1.15 are roughly 0.15 points above or below the 1.0 RRR value and are shaded with diagonal lines. The majority of these blocks are located in Cedar Rapids and Iowa City. Blocks with RRRs diverging from the 0.86 to 1.15 range are shaded in orange when they are below the range, and shaded in purple when they are above. Thus, orange blocks represent areas where the average RRR indicates relative low recovery. These blocks are located across Cedar Rapids. By contrast, purple blocks represent areas where the average RRR indicates relative high recovery. The high recovery blocks are located in Palo and in Cedar Rapids.

### 5.2. Recovery by Federal Program

To determine recovery differences between the programs, the household RRR measures are compared between HMGP and CDBG. The Mann-Whitney *U* Test was significant (*p* < 0.001), with a test statistic of 9172. The mean rank for HMGP was two times greater than the mean rank for CDBG. The test provides evidence that the mean RRR for CDBG (0.76) is significantly lower than for HMGP acquisitions (1.22). Thus, relative recovery differs between federal acquisition programs in both Cedar Rapids and Iowa City.

The CDBG distribution has a wider RRR range and a large standard deviation compared to the HMGP distribution (Table 5). Thus, the final dollar amounts for CDBG acquisitions were more likely to diverge from the appraisal values, while funds for HMGP acquisitions were more consistent. The lower RRR values and higher variability for CDBG funds may be due to difficulties in local level implementation and conflicting acquisition guidelines, which could result in partial and delayed funding from incomplete and longer application processes. HMGP acquisitions, on the other hand, have well documented and established procedures [63].

### 5.3. Recovery for Vulnerable Populations

Regression models were developed to understand the relationship between recovery and vulnerable populations. The OLS regression model is statistically significant and has an adjusted *R*-squared value of 0.746, which means the OLS model explains 74.6% of the variability in average RRR (Table 6). However, the Moran’s I test of the residuals shows a statistically significant value of −6.94 (*p* < 0.01). The results indicate negative spatial autocorrelation, which violates the assumption of independent observations in a traditional OLS regression. Therefore, we applied econometric modeling to account for spatial effects. The robust Lagrange multiplier is significant for both the lag and error tests, so we applied both models separately. Since the error and lag model incorporate the spatial autoregressive term, the OLS adjusted R-squared is no longer applicable to compare across all models [50]. Instead the AIC_c_ is used to compare and rank multiple regression models [64]. The error model had better fit than the lag model as determined by its more negative AIC_c_ value, and reduces the OLS AIC_c_ by half. Thus, the error model provides model is also included in Table 6.

Based on the OLS model, the percent Hispanic and percent elderly variables are statistically and negatively correlated with the average RRR variable (Table 7). Although the coefficients are relatively small, the negative relationship between these potentially vulnerable populations and recovery aligns with the literature on disaster recovery. The coefficients for percent Hispanic and percent elderly are identical in the OLS and error models, but increase in significance in the error model. Many factors contribute to the vulnerability and disproportionate recovery of elderly, including low social capital, limited mobility, and medical illness [65,66]. Hispanics are a large and diverse population group and many factors could potentially contribute to lower recoveries, including language barriers, recent migration, lack of local knowledge, and missing property-ownership documentation required for buyout funds [67,68].

The other social vulnerability variables did not have the expected relationships with the average RRR measure. The English proficiency variable changes from being non-significant in the OLS to being significant in the error model. The English proficiency variable has a low mean compared to a high standard deviation, which may be causing unstable variance in the model (see Table 4). In both models, percent Blacks and percent renters were not statistically significant. This might be a result of the small proportion of Blacks in the area, and the high correlation with renter and low median household income populations. The median household income in its natural log-form was not included in any of the models because of high negative pairwise correlations with the variables for percent Black, percent renter, and CDBG acquisitions. Collinearity diagnostics and condition index measures were acceptable for the remaining model variables.

The variables for HMGP acquisitions and average flood depth were both significant in the OLS and error models. The HMGP acquisitions variable has the largest standardized coefficient in the models, and has a positive relationship with average RRR. Thus, after controlling for other variables, the type of funding program is still a strong predictor of average RRR. However, the HMGP variable loses some predictive power as indicated by the smaller coefficient in the error model compared to the OLS model. The average depth was also significant, but negatively correlated with the average RRR in both the OLS and error models. Although flood depth is not a direct factor in assessing property acquisition cost, this variable was included to explore the relationship between level of flood severity and recovery. The flood depth was negatively correlated with average RRR, meaning that areas with greater flooding tended to have lower RRR values. 

Overall, the error model has improved model fit. However, the likelihood ratio test is still significant in the error model (Table 6), suggesting remaining specification problems. Applying the univariate Moran’s I on the error model residuals yields a statistic of −0.30, with a statistically significant pseudo *p*-value of 0.001. Thus, the spatial error model still does not fully account for spatial effects. This may be due to spatial heterogeneity, or spatially varying relationships, that is not being accounted for in our autoregressive model. In other words, the same housing attributes or acquisition attributes could create different housing values or acquisition cost in different parts of the study area [50]. Spatial nonstationarity can be remediated through geographically weighted regression (GWR) analysis [57]. However, GWR models with a sample size smaller than 160 are not viable [69,70].

## 6. Discussion

### 6.1. Recovery Differences by Federal Program

Implementing HMGP and CDBG acquisitions to meet local recovery objectives led to particular spatial patterns. For example, Cedar Rapids had both mitigation and neighborhood development objectives (see Table 2). Because HMGP acquisitions cannot have structural improvements, Cedar Rapids used CDBG funds to meet their development needs. As shown in Figure 1a, program restrictions in combination with local community objectives led to the spatial concentration of HMGP acquisitions along the river, which was designed as the Greenway Area. By contrast, CDBG acquisitions occur across the entire floodplain in both the Construction Area and the Neighborhood Revitalization Area. Similar patterns emerged in Iowa City.

Temporal discrepancies between HMGP and CDBG acquisitions also exist. In Cedar Rapids, the HMGP application was submitted in January 2009, the program was funded by September 2009, and the acquisitions were completed by April 2011 [44,71]. The CDBG application was submitted in 2009, the program was funded in March 2010, but the acquisitions were not completed until July 2014 [44,72]. In Iowa City, HMGP acquisitions were completed by July 2011 and CDBG acquisitions were completed by June 2013. In both cities, HMGP acquisitions were completed at least two years before CDBG acquisitions.

Many factors contributed to the temporal discrepancy of completed acquisitions by program. These factors may include political and bureaucratic procedures, communication between program managers and home-owners, community capacity, and competing mitigation plans [73]. In addition, requirements for environmental and planning reviews might have delayed distribution of federal grants. In New York City after Hurricane Sandy, a year passed before CDBG reimbursements for home repair became available [40]. Our results indicate that this temporal discrepancy also occurred for property acquisition funds. Thus, there are inequitable experiences between those with resources who are able to finance their own recovery, and those without resources who become entangled in a bureaucratic maze of regulatory reviews and slow recovery waiting for federal funds [40].

It is likely that homeowners waiting on CDBG acquisition funds had to combine other federal grants, subsidized loans, and personal savings to support immediate recovery needs. For example, Cedar Rapids buyout participants had limited affordable housing options, because the flood impacted the majority of the affordable housing stock within the city [74]. Our data show that hundreds of SBA subsidized-disaster loans and FEMA’s Individual Assistance grants were provided to individuals, including property acquisition participants. Some property acquisition participants may have received additional grants of up to $25,000 from the Replacement Housing Assistance program and up to $10,000 in supplemental housing payments [75]. Supplemental grants would have been especially important for individuals waiting on CDBG acquisition funds. However, the household RRR range of 0.09 to 2.02 includes dollar amounts as low as $3,000. Thus, it is difficult to determine the extent to which individuals with an RRR below 1.0 were able meet the remaining financial need through the other programs. Even so, this complicates the process of recovery for the households that are dealing with multiple programs and their varying requirements, potentially extending the time before receipt of funds.

Furthermore, the combination of aid from many different federal programs may have resulted in duplication of benefits not expected by homeowners [39]. In Hurricane Sandy, SBA loans became quickly available but in the long term this slowed CDBG reimbursements because of the additional verification for duplication of benefits [40]. Similar requirements likely delayed disbursement of CDBG funds in Cedar Rapids. If duplication of benefits was a common occurrence among homeowners waiting for CDBG funds, it may account for the lower mean RRR as compared to HMGP (see Table 5). In other words, both HMGP and CDBG participants potentially received similar funding totals, but with substantial differences in the timing of allocation.

### 6.2. Social Vulnerability and Recovery

The OLS and error regression models both predict average recovery rates in relation to social vulnerability indicators (Table 7). The relationship between neighborhoods with higher Hispanic populations and lower recovery rates was statistically significant. This finding is consistent with previous findings in the social vulnerability literature [10,28,29,58]. Additionally, neighborhoods with high elderly populations overlapped with areas of lower average recovery rates. The low average recovery rates associated with Hispanic and elderly populations have at least three potential effects. First, these groups are historically among the most vulnerable to hazards and often become worse off without full and timely compensation through recovery resources. Second, relocation effects for these populations are greater when support from local social systems are lost after relocation [6]. Finally, these populations (particularly those of low-income) may be unable to secure affordable housing outside of the floodplain, and thus remain at risk and vulnerable [73].

The distribution of acquisition resources aligns with neither the Rawls difference principle nor the libertarian principle. In this study, 10% of the acquisition participants had higher proportions of federal funds as indicated by the RRR values ranging from 1.2 to 2.02. It is unlikely that these households are among the most vulnerable because elderly and Hispanic populations correlated with lower RRRs. This is an inequitable distribution of federal funds under the Rawls difference principle [52], where inequity is defined as that which disproportionally favors well-off groups over those in unfavorable and vulnerable conditions [9,10,11,12]. The resource distribution also does not follow a libertarian principle, which would have been the case if all RRRs were near equivalent to 1.0 since that would be the numerical representation of free-market based resource allocation.

It is likely that acquisitions with RRR values substantially greater than 1.0 were achieved by individuals with social connections and the political and economic power to access higher proportions of federal funds [17,28,29]. These characteristics are not easily measurable, but could be influential in legal processes such as appraisal appeals. In Cedar Rapids, fifty-three out of the fifty-eight individuals who appealed their first appraisal were successful. Collectively, they obtained an increased appraisal from 107% pre-flooded value to 114% [43]. Appraisal appeal is a legal process necessitating City Council approval, and requiring energy, time, and resources from the homeowner including hiring a licensed appraiser [76]. Vulnerable populations, who often have limited access to political and economic resources, are less likely to appeal an appraisal successfully. Future research that can draw connections between social vulnerability and the individual characteristics of property acquisition participants is needed to better understand differential outcomes in recovery.

## 7. Conclusions

This research investigated the distribution of federal funds for property acquisitions in the recovery stage of a flood disaster. Federal funds for acquisition projects are an important resource to facilitate flood recovery and advance hazard mitigation. However, political and economic processes in property acquisition do not guarantee the equal recovery of all those involved. Our study finds that households in high social vulnerability areas were less likely to obtain full financial compensation, and endured longer periods before receiving acquisition funds. We also found that programmatic stipulations create distinct spatial distribution of acquisitions by federal program. These can lead to subsets of acquisitions that are prioritized and completed before others, and result in temporal inequalities. In particular, property acquisitions in areas of high elderly and Hispanic populations on average had lower recovery rates. Although low recovery rates might be an effect of duplication of benefits, the extreme high recovery rates may be due to political and social processes that allow advantaged individuals to obtain a disproportionate amount of the resources.

A key factor that emerged in the analysis was differences in the timing of acquisition funding between federal programs and potentially across vulnerable populations. This leads to a host of subsequent questions for future research. For example, how long was it before homeowners were able to purchase a replacement home? What are the connections between the timing and amount of funds for property acquisition with other aspects of recovery? Do inequalities or barriers in the acquisition process amplify throughout other aspects of recovery such as relocation? These questions need to be further investigated to understand long-term implications of property acquisition and recovery, and to make sound policy recommendations for emergency managers and planners involved in disaster recovery. Studies of environmental equity should also focus on temporal aspects along with procedural and distributive concerns.

## Figures and Tables

**Table 1:** Characteristics of study area.

Characteristics	Cedar Rapids	Palo	Iowa City
Total Population (Count)	125,951	941	67,067
Elderly (%)	12.90%	4.50%	7.60%
Minority (%)	12.53%	4.57%	14.62%
Linguistic Isolation Spanish (%)	6.40%	0%	15.50%
Tenure Owner-Occupied (%)	68.98%	91.46%	47.54%
Median Value of Owner-Occupied Housing (Dollars)	$123,200	$168,300	$171,600

**Table 2:** Property acquisition by city.

City	Acquired Properties	Appraisal Multiplier	Total Cost	Recovery Objectives
Cedar Rapids	1183	107%	$58.1 million	Non-structural & structural mitigation, rebuilding, housing development
Palo	12	110%	$2 million	Non-structural mitigation, rebuilding, economic development
Iowa City	93	112%	$22 million	Non-structural & structural mitigation

**Table 3:** Input geospatial data.

Input Data	Attributes	Sources
HMGP buyouts	Addresses and cost	FEMA
CDBG buyouts	Addresses and cost	IEDA
Flood depth grids	Peak depth in 2008	Iowa Flood Center
Demographics	Block group demographic values	2000 U.S. Census and TIGER Line
Assessed values, and parcels	Addresses, class, occupancy, and 2008 total value	Johnson County, Linn County, and Cedar Rapids assessors

**Table 4:** Descriptive statistics for model variables.

Continuous Variables	Mean	SD
** Average RRR	0.93	0.20
% Black	3.85	5.58
% Hispanic	2.62	2.63
% Elderly	19.05	9.08
% Renter	33.93	19.24
% Limited English Proficiency	0.97	1.37
Medium Household Income (Ln)	10.56	0.42
Population Density	996.35	827.03
Average Flood Depth	2.20	1.24
**Control Variables**	**Count**	
City Variable	25	
HMGP Acquisitions	6	
CDBG Acquisitions	14	
Both HMGP and CDBG	5	

** Dependent variable, Sample size = 25.

**Table 5:** Descriptive statistics for the relative recovery ratio.

Program	Sample Size	Mean	SD	Min	Max
HMGP	142	1.22	0.22	0.23	1.94
CDBG	931	0.76	0.31	0.09	2.02
Total	1073	0.82	0.34	0.09	2.02

**Table 6:** OLS and error model statistics.

OLS Model		Error Model	
Adj. *R*-squared	0.746	*R*-squared	0.811
Log Likelihood	28.31	Log Likelihood	42.03
AIC_c_	−34.61	AIC_c_	−62.07
Schwarz Criterion	−21.21	Schwarz Criterion	−48.66
Breusch-Pagan	17.62	Breusch-Pagan	11.26
Moran’s I (Error)	** −6.94	Likelihood Ratio Test	** 27.45
LM Lag	0.26		
Robust LM Lag	* 20.89		
LM Error	** 32.73		
Robust LM Error	** 32.99		

* *p* < 0.05; ** *p* < 0.01.

**Table 7:** Standardized coefficients for OLS and error models.

Model Variable	OLS	Error
Constant	** 1.20	** 1.21
% Black	0.01	0.01
% Hispanic	* −0.03	** −0.03
% Elderly	* −0.01	** −0.01
% Renter	0.00	0.00
% Limited English Proficiency	0.05	* 0.05
Population Density	0.00	0.00
Average Flood Depth	* −0.05	* −0.04
City Dummy	0.13	0.11
HMGP Acquisitions	* 0.26	* 0.21
CDBG Acquisitions	−0.06	−0.07
Lambda	–	0.42

* *p* < 0.05; ** *p* < 0.01.

## Abbreviations

The following abbreviations are used in this manuscript:

HMGP	Hazard Mitigation Grant Program
CDBG	Community Development Block Grant
FEMA	Federal Emergency Management Agency
SBA	Small Business Administration
IEDA	Iowa Economic Development Authority
RRR	Relative Recovery Ratio
OLS	Ordinary Least Squares
AIC_c_	Adjusted Akaike’s Information Criterion
SD	Standard Deviations
GWR	Geographically weighted regression
